# Efficacy of neoadjuvant immunochemotherapy in locally advanced esophageal squamous cell carcinoma: a prospective cohort study with propensity-score matching

**DOI:** 10.3389/fimmu.2025.1711095

**Published:** 2026-01-06

**Authors:** Shifa Zhang, Anhao Hu, Jiuhe Sun, Yutao Wei, Jishan Zhang, Haibo Cai, Gongchao Wang

**Affiliations:** 1 Department of Thoracic Surgery, Shandong Provincial Hospital, Shandong University, Jinan, Shandong, China; 2 Department of Thoracic Surgery, Jining No.1 People’s Hospital, Affiliated Jining No.1 People’s Hospital of Jining Medical University, Jining, Shandong, China; 3 Department of Clinical Medicine, Jining Medical University, Jining, China

**Keywords:** esophageal squamous cell carcinoma, neoadjuvant immunochemotherapy, PD-1 blockade, propensity-score matching, overall survival, progression-free survival

## Abstract

**Background:**

This study compared the efficacy of neoadjuvant immunochemotherapy (nICT) followed by surgery versus upfront surgery for locally advanced esophageal squamous cell carcinoma (ESCC).

**Methods:**

In this prospective controlled trial (2020-2025), 623 stage II–IIIB ESCC patients were included; 192 received nICT (cisplatin, nab-paclitaxel, sintilimab) before surgery, and 431 underwent direct surgery. Propensity-score matching (PSM) and overlap weighting were used to adjust for baseline confounders.

**Results:**

After PSM (144 pairs), the nICT group showed significantly improved progression-free survival (PFS; HR = 0.31, p<0.001) and overall survival (OS; HR = 0.42, p=0.002) compared to the upfront surgery group. The nICT group also had higher 1-/3-year PFS (88.8%/84.3% vs. 68.1%/52.8%) and OS rates (94.8%/84.7% vs. 89.6%/65.2%). Sensitivity analysis using overlap weighting confirmed these robust findings (PFS: HR = 0.37, p<0.001; OS: HR = 0.62, p=0.033).

**Conclusions:**

For locally advanced ESCC, neoadjuvant immunochemotherapy significantly improves both PFS and OS compared to upfront surgery, establishing it as a highly effective treatment strategy.

## Introduction

Esophageal cancer is one of the most common malignant tumors in China, with squamous cell carcinoma being the predominant histological type, accounting for more than 90% of cases (1). The majority of patients are diagnosed at a locally advanced stage, at which point the tumor often invades surrounding tissues or involves regional lymph node metastasis. This makes direct surgical resection challenging and results in poor prognosis. Therefore, neoadjuvant therapy before surgery is of great significance in improving patient outcomes. Currently, neoadjuvant therapies for esophageal cancer include neoadjuvant chemotherapy, neoadjuvant radiochemotherapy, neoadjuvant immunotherapy, and neoadjuvant radiochemotherapy combined with immunotherapy. Among these, neoadjuvant radiochemotherapy is considered the standard neoadjuvant treatment, significantly improving the resection rate and long-term survival of patients (2). However, due to the increased surgical difficulty and subsequent higher risk of postoperative complications associated with radiochemotherapy, its application in Chinese patients with esophageal cancer is relatively limited. Thus, there is an urgent need to explore an alternative treatment that is as effective as neoadjuvant radiochemotherapy but has fewer side effects and less impact on surgical difficulty.

In recent years, immunotherapy, represented by PD-1 inhibitors, has achieved significant success in Phase III clinical trials for first-line treatment of esophageal cancer (3–5), providing a theoretical basis for the combination of immunotherapy and chemotherapy as a neoadjuvant treatment for esophageal cancer. For instance, a study by Liu J et al. (6) demonstrated that the combination of camrelizumab and chemotherapy as neoadjuvant treatment for locally advanced esophageal squamous cell carcinoma resulted in pathological complete response in 20 patients (20/51, 39.2%), with 34 patients (34/51, 56.7%) experiencing grade 3 or higher adverse reactions. Notably, there were no deaths during hospitalization or within 30 or 90 days postoperatively. Additionally, 98% of patients (50/51) achieved R0 resection without increased surgical difficulty. These findings suggest that the combination of immunotherapy and chemotherapy may be an effective neoadjuvant treatment option.

With the rise of immunotherapy for advanced esophageal cancer, its application has gradually expanded from first-line standard treatment to perioperative exploration. In 2022, the Chinese Society of Clinical Oncology (CSCO) published guidelines for the diagnosis and treatment of esophageal cancer, recommending that some patients with thoracic esophageal cancer could adopt chemotherapy combined with immunotherapy as a neoadjuvant treatment (7). However, the current evidence for this approach is relatively limited, with most studies still in the clinical trial phase.

Most contemporary studies of neoadjuvant immunochemotherapy for esophageal cancer are single-arm, small-sample investigations with short follow-up. Since September 2020, the Department of Thoracic Surgery at Jining First People’s Hospital has conducted a single-center, non-randomized phase II–III trial comparing pre-operative immunochemotherapy followed by surgery versus upfront surgery in patients with locally advanced esophageal squamous cell carcinoma. Building upon a preliminary analysis of an early patient subset (8), this final analysis of the complete cohort (n=623) leverages a significantly larger sample size and propensity score matching to achieve superior baseline balance, thereby yielding more credible and conclusive results that significantly advance the previous findings. In the present trial, treatment allocation was determined by patients and their families after full informed consent, resulting in marked baseline imbalances between groups. To address this, propensity-score matching (PSM) and overlap weighting (OW) were applied to the cohort of patients with stage IIB–IIIB ESCC enrolled between 2020 and 2025, allowing comparison of progression-free survival (PFS) and overall survival (OS) between the immunochemotherapy-plus-surgery and upfront-surgery arms, with the goal of providing high-level evidence for neoadjuvant immunochemotherapy in esophageal cancer.

## Methods

### General data

**Figure 1:**
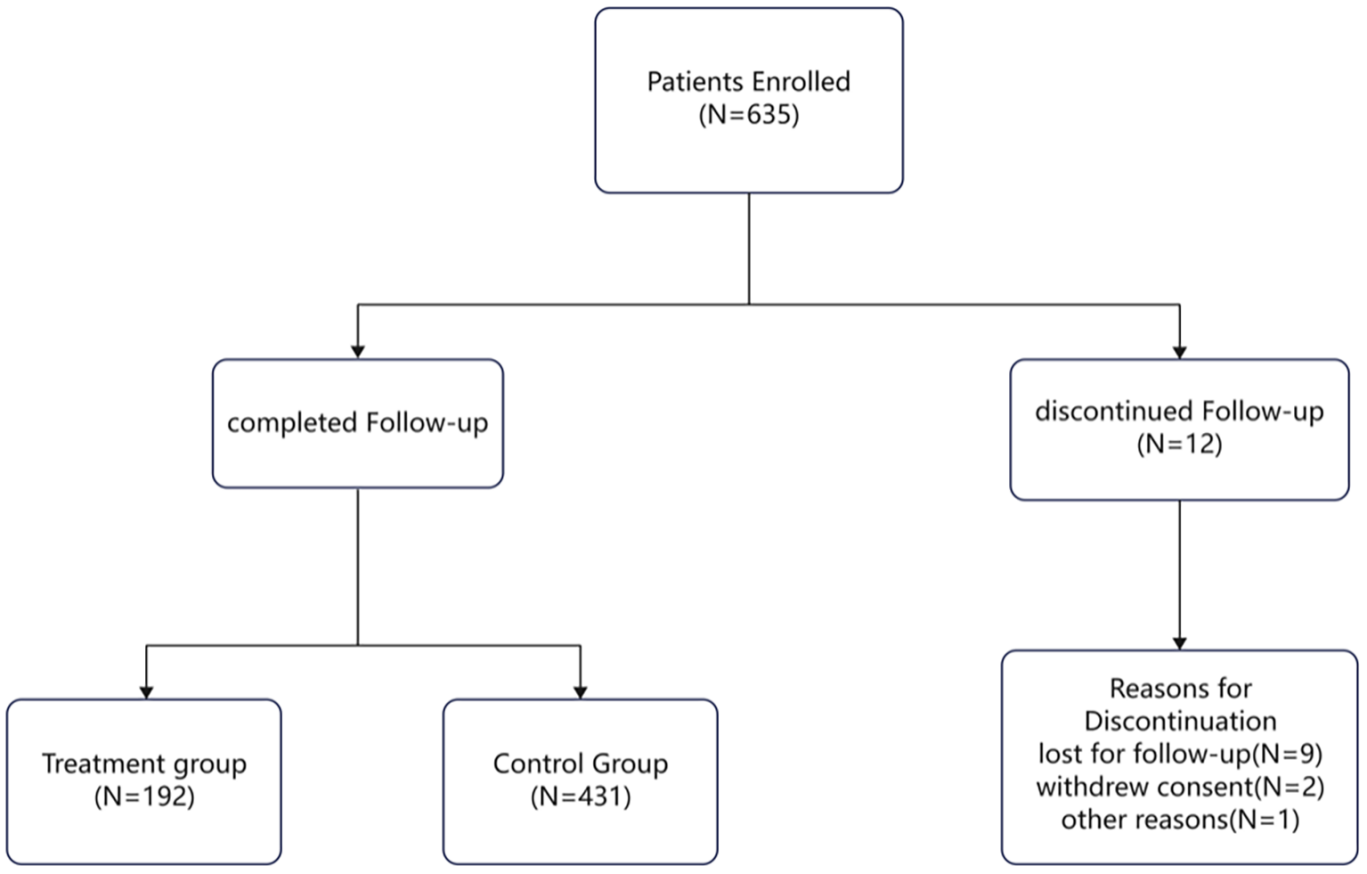
Flowchart of patients. A total of 635 patients were enrolled in the study. Among them, 623 cases completed follow-up and provided valid data, while 12 cases were discontinued. Of these dropouts, 9 were lost to follow-up, 2 withdrew consent, and 1 was transferred to another hospital.

All data in this study were derived from a non-randomized, single-center, prospective controlled clinical trial conducted at our hospital from January 2020 to March 2025. A total of 623 patients with stage II–IIIB esophageal squamous cell carcinoma (ESCC) were ultimately included in the study, Detailed information is provided in **Figure 1**. Among them, 347 patients (55.7%) were aged ≥65 years and 276 (44.3%) were <65 years; 451 patients (72.4%) were male and 172 (27.6%) were female.192 patients received neoadjuvant immunochemotherapy followed by surgery, whereas 431 patients underwent upfront surgery. Because assignment was non-random, baseline characteristics—especially T and N staging—were imbalanced between groups. Propensity-score matching (PSM) and overlap weighting (OW) were therefore applied to achieve covariate balance before survival analyses were performed. Baseline characteristics are summarized in **Table 1**.

**Table 1:** General clinical characteristics of patients prior to propensity score matching.

Variables	Total (N = 623)	NO (N = 431)	YES (N = 192)	P value
Sex (%)				0.699
female	172 (27.6)	117 (27.1)	55 (28.6)	
male	451 (72.4)	314 (72.9)	137 (71.4)	
Age, Mean ± SD	65.22 ± 7.78	64.55 ± 7.96	66.73 ± 7.16	0.001
Smoking (%)				0.060
yes	267 (42.9)	174 (40.4)	93 (48.4)	
no	356 (57.1)	257 (59.6)	99 (51.6)	
Alcohol consumption (%)				0.016
no	353 (56.7)	258 (59.9)	95 (49.5)	
yes	270 (43.3)	173 (40.1)	97 (50.5)	
Tumor location (%)				0.012
Upper	83 (13.3)	69 (16)	14 (7.3)	
Middle	413 (66.3)	278 (64.5)	135 (70.3)	
Lower	127 (20.4)	84 (19.5)	43 (22.4)	
Clinical T stage (%)				< 0.001
T3-T4	543 (87.2)	359 (83.3)	184 (95.8)	
T1-T2	80 (12.8)	72 (16.7)	8 (4.2)	
Clinical N stage (%)				< 0.001
N0	401 (64.4)	322 (74.7)	79 (41.1)	
N1-N2	222 (35.6)	109 (25.3)	113 (58.9)	
Operation time, Median (Q1,Q3)	260.00 (225.00,300.0)	270.00 (240.00, 300.00)	240.00 (210.00, 274.25)	< 0.001
Blood loss, Median (Q1,Q3)	100.00 (100.00,100.0)	100.00 (100.00, 100.00)	100.00 (100.00, 100.00)	< 0.001
Differentiation (%)				< 0.001
Poor	167 (26.8)	134 (31.1)	33 (17.2)	
Moderate	338 (54.3)	205 (47.6)	133 (69.3)	
Well	118 (18.9)	92 (21.3)	26 (13.5)	
Margin (%)				0.106
Negative	590 (94.7)	404 (93.7)	186 (96.9)	
Positive	33 (5.3)	27 (6.3)	6 (3.1)	
Adjuvant Treatment(%)				< 0.001
Observation or Chemotherapy	471 (75.6)	346 (80.3)	125 (65.1)	
Radiotherapy or Chemoradiotherapy	152 (24.4)	85 (19.7)	67 (34.9)	
pTNM stage, Median (Q1,Q3)	2.50 (2.00, 3.50)	3.00 (2.50, 3.50)	2.00 (1.50, 3.50)	< 0.001

Because this was a non-randomized study, the two groups differed in baseline clinical stage. The neoadjuvant cohort contained significantly more advanced tumors (cT3–T4 and cN1–N2) than the upfront-surgery cohort. pTNM: pathologic TNM stage.

All participants were enrolled under the clinical-trial registration number ChiCTR2100044427. The study protocol was approved by the Ethics Committee of Jining First People’s Hospital. Before enrollment, every patient (or their legal guardian) received detailed information regarding potential benefits and risks and provided written informed consent.

### Inclusion criteria

Patients aged 40–80 years, regardless of gender. 2. Histologically and radiologically confirmed, untreated esophageal squamous cell carcinoma. 3. Staging according to the 8th edition of the American Joint Committee on Cancer (AJCC) clinical staging for esophageal squamous cell carcinoma, classified as cT1-3N1-2M0 or cT2-3N0M0 (Stage II/III). 4. Pre-treatment assessment deemed suitable for R0 surgical resection. 5. Presence of measurable lesions according to the Response Evaluation Criteria in Solid Tumors version 1.1 (RECIST v1.1). 6. Eastern Cooperative Oncology Group (ECOG) performance status score of 0–2. 7. Voluntary participation in the study with good compliance and ability to complete safety and survival follow-up. Written informed consent was obtained from all patients and their legal guardians prior to enrollment.

### Exclusion criteria

Previous exposure to radiotherapy, chemotherapy, or other anti-tumor treatments. 2.Prior treatment with other PD-1/PD-L1 inhibitors or allergy to any component of PD-1 inhibitors. 3. Presence of active autoimmune diseases or history thereof (e.g., autoimmune hepatitis, interstitial pneumonia, pneumoconiosis, radiation pneumonitis, uveitis, enteritis, hepatitis, hypophysitis, vasculitis, nephritis, hyperthyroidism, hypothyroidism, etc.). 4. Uncontrolled cardiovascular diseases, including but not limited to: New York Heart Association (NYHA) functional class II or higher heart failure, unstable angina, myocardial infarction within the past year, or arrhythmias requiring treatment. 5.Significant coagulation abnormalities (e.g., prothrombin time >16 seconds, activated partial thromboplastin time >43 seconds, thrombin time >21 seconds, fibrinogen >2 g/L), bleeding tendency, or undergoing thrombolytic therapy. 6. Active bleeding caused by diseases such as active peptic ulcers in the stomach and duodenum, portal hypertension, or other conditions that may lead to gastrointestinal bleeding or perforation within the past 3 months. 7.Suspected active infection or unexplained fever (temperature >38.5°C). 8. Congenital or acquired immunodeficiency, such as HIV infection, active hepatitis B or C. 9.Other factors deemed by the investigators to necessitate exclusion, such as other severe diseases (including psychiatric conditions), need for concomitant treatments, significant laboratory abnormalities, or social/family factors that may affect patient safety or interfere with data and sample collection.

### Withdrawal criteria

1.Voluntary withdrawal from the study by the patient or their legal guardian. 2. Unexpected rapid disease progression or radiologically confirmed rapid progression. 3. Continued participation in the study deemed to offer no anticipated benefit to the patient. 4. Grade 3 or higher adverse reactions during immunotherapy combined with chemotherapy that do not resolve with treatment. 5. Treatment delay exceeding 2 weeks due to any reason. 6. Withdrawal deemed necessary by the investigators based on the overall condition of the patient. 7. All patients who withdraw from the study should be followed up according to the study protocol, and the follow-up results should be documented, unless the patient revokes the informed consent and refuses follow-up.

### Grouping method

In this study, due to ethical constraints—specifically, the principle that randomization is impermissible when an established effective therapy exists—a randomized controlled trial could not be implemented. Consequently, a non-randomized design was adopted. Treatment assignment was determined by voluntary choice: after receiving comprehensive information, each patient (together with his or her family) selected the treatment arm. To minimize possible selection bias or undue influence, the physician who provided the counseling was, in principle, not the patient’s attending doctor.

### Treatment protocol

All 192 patients received at least two cycles of the following regimen: cisplatin (75 mg/m², intravenous infusion, 21-day cycle), nab-paclitaxel (260 mg/m², intravenous infusion, 21-day cycle), and sintilimab (200 mg, intravenous infusion on Day 3 of chemotherapy, 21-day cycle). After 2 cycles of combined chemotherapy and immunotherapy, surgery was performed 4–6 weeks later. The surgical procedure was minimally invasive esophagectomy (MIE) via a three-incision thoracoabdominal approach. Postoperative adjuvant chemotherapy and immunotherapy (2 cycles) were determined based on the final pathological findings. Follow-up examinations included contrast-enhanced chest CT, tumor markers, abdominal ultrasound, and cervical lymph node assessment every 3 months.

### Follow-up

A combined strategy of active follow-up and passive surveillance was used. Follow-up time was calculated from the date treatment was initiated. The median follow-up duration was 28 months; the evaluable follow-up duration was 32 months, with data censored on 31 March 2025. The schedule adhered to current clinical-practice guidelines: every 3 months for the first 2 years, every 6 months from year 3 to year 5, and annually thereafter. Patients who missed visits were contacted by telephone or mail, and information was also sought from the patient or first-degree relatives to minimize bias from censored data.

The primary end-points were overall survival (OS) and progression-free survival (PFS). OS was defined as the interval from treatment initiation to death from any cause. PFS was defined as the interval from treatment initiation to documented disease progression (per RECIST 1.1) or death from any cause, whichever occurred first.

### Statistics

#### Statistical analysis

Statistical analyses were performed with R software (version 4.4.1). Continuous variables that followed or approximately followed a normal distribution are presented as mean ± SD; comparisons between groups were made with the independent-samples t-test.Categorical variables are expressed as frequencies (percentages); group comparisons were performed with the χ² test or Fisher’s exact test, as appropriate. Overall survival (OS) and progression-free survival (PFS) were visualized with Kaplan–Meier curves and compared across treatment groups with the log-rank test. Hazard ratios (HRs) and 95% confidence intervals (CIs) were estimated with Cox proportional hazards regression. Because this is a non-randomized study, we used propensity score matching (PSM) implemented with the R package MatchIt to control for baseline confounders. Matching was 1:1 without replacement, with a caliper width of 0.1 (on the logit scale). The following variables were included in the propensity model: sex, age, smoking, alcohol consumption, primary tumor site, clinical T stage, clinical N stage, operative time, intra-operative blood loss, histologic differentiation, surgical margin status, and post-operative therapy. In addition, inverse-probability-of-treatment weighting (IPTW) and overlap weighting were applied as sensitivity analyses, using the same covariate set. The weighting scheme that achieved the best post-weighting balance (standardized mean difference, SMD < 0.1 for all covariates) was selected for final analyses. In the weighted cohorts, continuous variables were compared with the weighted t-test, categorical variables with the Rao–Scott adjusted χ² test, and survival curves with the weighted log-rank test. Weighted Cox regression was used to estimate HRs and 95% CIs. Balance was assessed with SMDs; an SMD < 0.1 after weighting was considered indicative of good balance. All tests were two-sided, and P < 0.05 was considered statistically significant.

## Results

### Comparison of baseline characteristics and survival outcomes

#### General clinical data

A total of 623 patients were ultimately included in the study: 192 in the neoadjuvant group and 431 in the surgery-only group. Because this was a non-randomized study, the two cohorts differed in baseline clinical stage. Patients in the neoadjuvant group had a higher proportion of T3–T4 disease (95.8% vs. 88.3%) and N1–N2 nodal involvement (58.9% vs. 25.3%) than those in the surgery-only group. Imbalances were also observed in age, alcohol consumption, tumor location, operative time, intra-operative blood loss, and post-operative therapy (**Table 1**).

### Survival analysis of progression-free survival

Univariate analysis showed that neoadjuvant therapy (p < 0.001), operative time (p < 0.001), and tumor differentiation (p < 0.001) were all significant factors influencing postoperative recurrence in esophageal cancer patients (details in **Table 2**). To clarify the independent effect of each factor, multivariable Cox proportional-hazards regression was performed. Neoadjuvant therapy significantly reduced the risk of recurrence (HR = 0.33, 95% CI: 0.25–0.51, p < 0.001), corresponding to a 67% risk reduction. Compared with poor differentiation, both well differentiation (HR = 0.41, 95% CI: 0.26–0.63, p < 0.001) and moderate differentiation (HR = 0.75, 95% CI: 0.56–1.01, p = 0.058) were protective, lowering recurrence risk by 59% and 25%, respectively. Additionally, operative time emerged as an independent risk factor (HR = 1.01, 95% CI: 1.00–1.01, p < 0.001), with each additional minute increasing recurrence risk by 1% (**Table 3**).

**Table 2:** Univariate analysis of postoperative progression-free survival (PFS) in esophageal cancer.

Variable	Estimate	Std.error	Statistic	HR(95%CI)	p
Neoadjuvant					
No	0.000			reference	
Yes	-1.029	0.200	-5.147	0.357 (0.242, 0.529)	<0.001
Sex					
Female	0.000			reference	
Male	0.052	0.152	0.341	1.053 (0.782, 1.419)	0.733
Age	-0.007	0.009	-0.854	0.993 (0.976, 1.009)	0.393
Smoking					
No	0.000			reference	
Yes	0.160	0.138	1.162	1.174 (0.896, 1.537)	0.245
Alcohol consumption					
No	0.000			reference	
Yes	0.060	0.136	0.442	1.062 (0.814, 1.386)	0.658
Tumor location					
Upper	0.000			reference	
Middle	-0.181	0.190	-0.950	0.835 (0.575, 1.212)	0.342
Lower	-0.276	0.235	-1.176	0.759 (0.479, 1.202)	0.240
Clinical T stage					
T3-T4	0.000			reference	
T1-T2	-0.362	0.210	-1.725	0.696 (0.462, 1.050)	0.084
Clinical N stage					
N0	0.000			reference	
N1-N2	0.185	0.140	1.322	1.203 (0.915, 1.582)	0.186
Operation time	0.006	0.001	6.067	1.006 (1.004, 1.009)	<0.001
Blood loss	0.000	0.000	0.634	1.000 (0.999, 1.001)	0.526
Differentiation					
Poor	0.000			reference	
Moderate	-0.522	0.147	-3.565	0.593 (0.445, 0.790)	<0.001
Well	-0.991	0.220	-4.501	0.371 (0.241, 0.572)	<0.001
Margin					
Negative	0.000			reference	
Positive	0.388	0.268	1.451	1.475 (0.873, 2.492)	0.147
Adjuvant Treatment					
Observation or Chemotherapy	0.000			reference	
Radiotherapy or Chemoradiotherapy	-0.118	0.163	-0.719	0.889 (0.646, 1.225)	0.472

Univariate analysis of postoperative recurrence in esophageal cancer identified neoadjuvant therapy (p < 0.001), operative time (p < 0.001), tumor differentiation (p < 0.001), and pathologic TNM stage (p < 0.001) as independent influencing factors.

**Table 3:** Multivariate analysis of postoperative progression-free survival (PFS) in esophageal cancer.

Variable	Estimate	Std.error	Statistic	HR(95%CI)	p
Neoadjuvant					
No	0.00			reference	
Yes	-1.10	0.22	-5.05	0.33 (0.22, 0.51)	<0.001
Sex					
Female	0.00			reference	
Male	-0.32	0.21	-1.53	0.73 (0.49, 1.09)	0.126
Age	-0.00	0.01	-0.20	1.00 (0.98, 1.02)	0.844
Smoking					
No	0.00			reference	
vYes	0.13	0.19	0.66	1.13 (0.78, 1.64)	0.510
Alcohol consumption					
No	0.00			reference	
Yes	0.18	0.17	1.07	1.20 (0.86, 1.67)	0.283
Tumor location					
Upper	0.00			reference	
Middle	-0.08	0.19	-0.42	0.92 (0.63, 1.35)	0.675
Lower	-0.30	0.24	-1.24	0.74 (0.46, 1.19)	0.217
Clinical T stage					
T3-T4	0.00			reference	
T1-T2	-0.30	0.22	-1.37	0.74 (0.48, 1.14)	0.170
Clinical N stage					
N0	0.00			reference	
N1-N2	0.41	0.15	2.72	1.51 (1.12, 2.03)	0.006
Operation time	0.01	0.00	4.63	1.01 (1.00, 1.01)	<0.001
Blood loss	-0.00	0.00	-0.29	1.00 (1.00, 1.00)	0.771
Differentiation					
Poor	0.00			reference	
Moderate	-0.29	0.15	-1.90	0.75 (0.56, 1.01)	0.058
Well	-0.89	0.22	-3.98	0.41 (0.26, 0.63)	<0.001
Margin					
Negative	0.00			reference	
Positive	0.18	0.28	0.65	1.20 (0.70, 2.06)	0.517
Adjuvant Treatment					
Observation or Chemotherapy	0.00			reference	
Radiotherapy or Chemoradiotherapy	0.02	0.17	0.14	1.02 (0.73, 1.43)	0.892

Multivariate Cox analysis showed that receiving neoadjuvant therapy was an independent protective factor against postoperative recurrence. Compared with poorly differentiated tumors, patients with well- or moderately differentiated tumors had a significantly lower risk of recurrence. Higher clinical N stage was an independent risk factor for postoperative recurrence.

**Figure 2:**
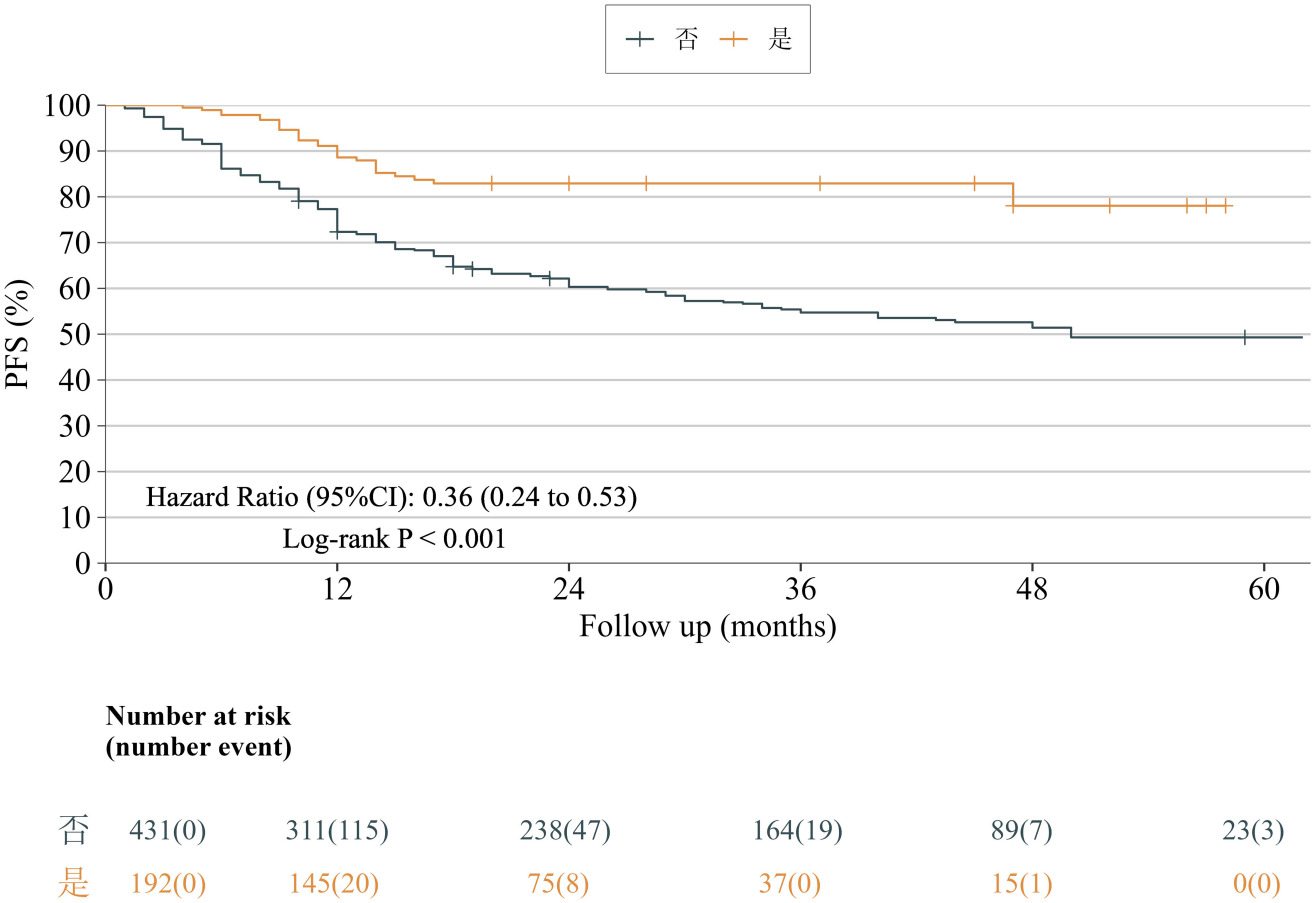
Kaplan–Meier curves of progression-free survival (PFS) for patients in the neoadjuvant-therapy group versus the direct-surgery group.

Kaplan–Meier analysis demonstrated a statistically significant difference in PFS between the neoadjuvant-therapy and direct-surgery groups (HR = 0.36, 95% CI: 0.24–0.53, p < 0.001), corresponding to a 64% reduction in the risk of disease progression or death. The median PFS was 50 months in the direct-surgery group, whereas the median PFS in the neoadjuvant-therapy group had not been reached during follow-up (**Figure 2**).

### Survival analysis of overall survival

Univariate analysis of overall survival (OS) after esophagectomy identified neoadjuvant therapy (p = 0.002), tumor differentiation (p < 0.001), operative time (p < 0.001), and clinical T stage (p = 0.049) as significant predictors of postoperative mortality; detailed statistics are provided in **Table 4**.

**Table 4:** Univariate analysis of postoperative overall survival (OS) in esophageal cancer.

Variable	Estimate	Std.error	Statistic	HR(95%CI)	p
neoadjuvant					
No	0.000			reference	
Yes	-0.677	0.218	-3.113	0.508 (0.332, 0.778)	0.002
Sex					
Female	0.000			reference	
Male	0.148	0.177	0.832	1.159 (0.819, 1.641)	0.405
Age	0.001	0.010	0.089	1.001 (0.982, 1.020)	0.929
Smoking					
No	0.000			reference	
Yes	0.125	0.155	0.807	1.134 (0.836, 1.537)	0.420
Alcohol consumption					
No	0.000			reference	
Yes	-0.068	0.154	-0.443	0.934 (0.690, 1.264)	0.658
Tumor location					
Upper	0.000			reference	
Middle	-0.124	0.214	-0.578	0.884 (0.581, 1.343)	0.563
lower	-0.236	0.270	-0.872	0.790 (0.465, 1.342)	0.383
Clinical T stage					
T3-T4	0.000			reference	
T1-T2	-0.460	0.234	-1.964	0.631 (0.399, 0.999)	0.049
Clinical N stage					
N0	0.000			reference	
N1	0.085	0.163	0.521	1.089 (0.791, 1.499)	0.603
Operation time	0.006	0.001	5.366	1.006 (1.004, 1.009)	<0.001
Blood loss	0.001	0.000	1.448	1.001 (1.000, 1.001)	0.148
Differentiation					
Poor	0.000			reference	
Moderate	-0.788	0.163	-4.838	0.455 (0.330, 0.626)	<0.001
Well	-1.208	0.244	-4.952	0.299 (0.185, 0.482)	<0.001
Margin					
Negative	0.000			reference	
Positive	0.407	0.300	1.359	1.503 (0.835, 2.704)	0.174
Adjuvant Treatment					
Observation or Chemotherapy	0.000			reference	
Radiotherapy or Chemoradiotherapy	-0.137	0.187	-0.730	0.872 (0.604, 1.259)	0.466

Univariate analysis of postoperative mortality in esophageal cancer identified neoadjuvant therapy (p = 0.002), tumor differentiation (p < 0.001), operative time (p < 0.001), and clinical T stage (p = 0.049) as independent influencing factors.

Multivariable Cox proportional-hazards regression showed that neoadjuvant therapy was associated with a significantly lower risk of death (HR = 0.58, p = 0.026), corresponding to a 42% reduction in mortality. Compared with poorly differentiated tumors, well-differentiated (HR = 0.33, p < 0.001) and moderately differentiated (HR = 0.54, p < 0.001) tumors were also protective, decreasing death risk by 67% and 46%, respectively. Operative time, modeled as a continuous variable, remained an independent predictor of OS (HR = 1.01, p < 0.001). Although the hazard ratio is close to unity, the unit is one minute; thus, each additional minute of surgery increased mortality risk by approximately 1%—a small but clinically meaningful effect. Detailed risk estimates are listed in **Table 5**.

**Table 5:** Multivariate analysis of postoperative overall survival (OS) in esophageal cancer.

Variable	Estimate	Std.error	Statistic	HR(95%CI)	p
Neoadjuvant					
No	0.00			reference	
Yes	-0.54	0.24	-2.23	0.58 (0.36, 0.94)	0.026
Sex					
Female	0.00			reference	
Male	-0.12	0.23	-0.51	0.89 (0.56, 1.40)	0.612
Age	0.00	0.01	0.23	1.00 (0.98, 1.02)	0.818
Smoking					
No	0.00			reference	
Yes	0.12	0.21	0.56	1.13 (0.74, 1.70)	0.575
Alcohol consumption					
No	0.00			reference	
Yes	-0.09	0.19	-0.46	0.92 (0.63, 1.33)	0.644
Tumor location					
Upper	0.00			reference	
Middle	-0.03	0.22	-0.15	0.97 (0.63, 1.49)	0.885
Lower	-0.28	0.28	-1.00	0.76 (0.44, 1.30)	0.318
Clinical T stage					
T3-T4	0.00			reference	
T1-T2	-0.31	0.24	-1.28	0.73 (0.45, 1.18)	0.201
Clinical N stage					
N0	0.00			reference	
N1	0.17	0.18	0.93	1.18 (0.83, 1.67)	0.352
Operation time	0.01	0.00	4.17	1.01 (1.00, 1.01)	<0.001
Blood loss	0.00	0.00	0.47	1.00 (1.00, 1.00)	0.639
Differentiation					
Poor	0.00			reference	
Moderate	-0.62	0.17	-3.62	0.54 (0.39, 0.75)	<0.001
Well	-1.09	0.25	-4.39	0.33 (0.21, 0.55)	<0.001
Margin					
Negative	0.00			reference	
Positive	0.16	0.31	0.51	1.17 (0.64, 2.15)	0.612
Adjuvant Treatment					
Observation or Chemotherapy	0.00			reference	
Radiotherapy or Chemoradiotherapy	-0.05	0.20	-0.27	0.95 (0.64, 1.40)	0.785

Multivariable Cox analysis of postoperative mortality in esophageal cancer showed that neoadjuvant therapy was associated with a hazard ratio (HR) of 0.58 (p = 0.026), corresponding to a 42% reduction in the risk of death compared with patients who did not receive this treatment. Well- and moderately differentiated tumors were also protective relative to poorly differentiated tumors (HR = 0.33, p < 0.001; HR = 0.54, p < 0.001), lowering mortality risk by 67% and 46%, respectively.

**Figure 3:**
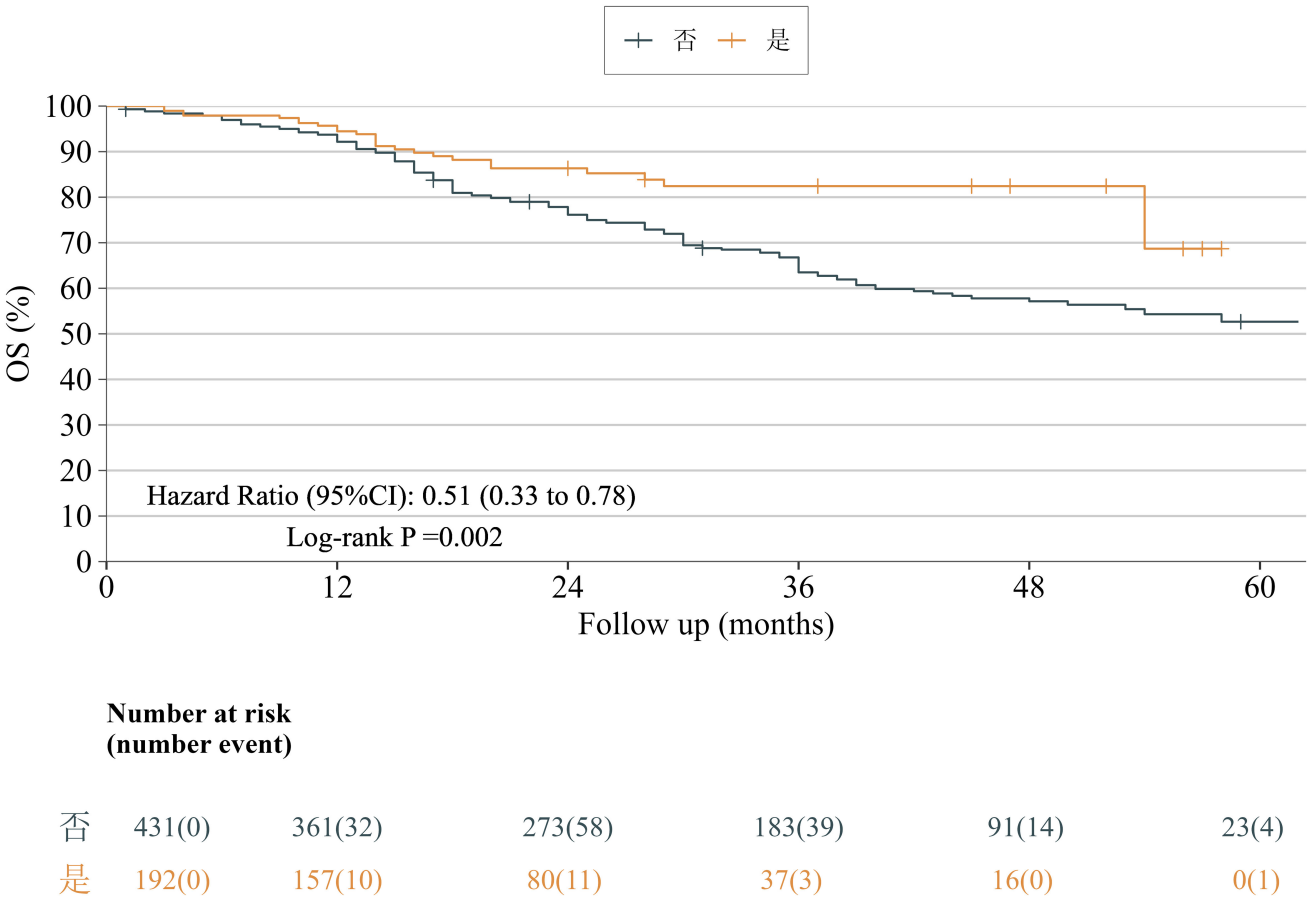
Overall survival curves for the neoadjuvant and non-neoadjuvant groups. Kaplan–Meier survival curves revealed a significant difference in overall survival between patients who received neoadjuvant therapy and those who did not (log-rank P = 0.002).

Kaplan–Meier analysis demonstrated significantly superior overall survival in the neoadjuvant-therapy group versus the direct-surgery group (HR = 0.51, 95% CI: 0.33–0.78, p = 0.002), translating to a 49% reduction in death risk. The median OS was 63 months in the direct-surgery group, whereas it had not been reached in the neoadjuvant-therapy group during the follow-up period (**Figure 3**).

### Survival analysis after propensity score matching and overlap weighting

Before propensity-score matching, the two cohorts were comparable with respect to smoking status, sex, and resection-margin status (all P > 0.05). However, statistically significant imbalances were observed for post-operative therapy (P < 0.001), age (P = 0.001), alcohol consumption (P = 0.016), tumor location (P = 0.012), clinical T stage (P < 0.001), clinical N stage (P < 0.001), tumor differentiation (P < 0.001), intra-operative blood loss (P < 0.001), pathologic TNM stage (P < 0.001), and operative time (P < 0.001), detailed statistics are provided in **Table 1**.

### Survival analysis after PSM

To control confounding, propensity-score matching (PSM) was performed with a caliper of 0.1 and a 1:1 ratio. Matching covariates included sex, age, smoking, alcohol consumption, tumor location (T-site), clinical T stage, clinical N stage, operative time, intra-operative blood loss, differentiation, margin status, and post-operative therapy. Because pathologic TNM stage acts as a mediator (accounting for roughly two-thirds of the effect) between neoadjuvant therapy and prognosis, it was excluded from the matching set.

**Figure 4:**
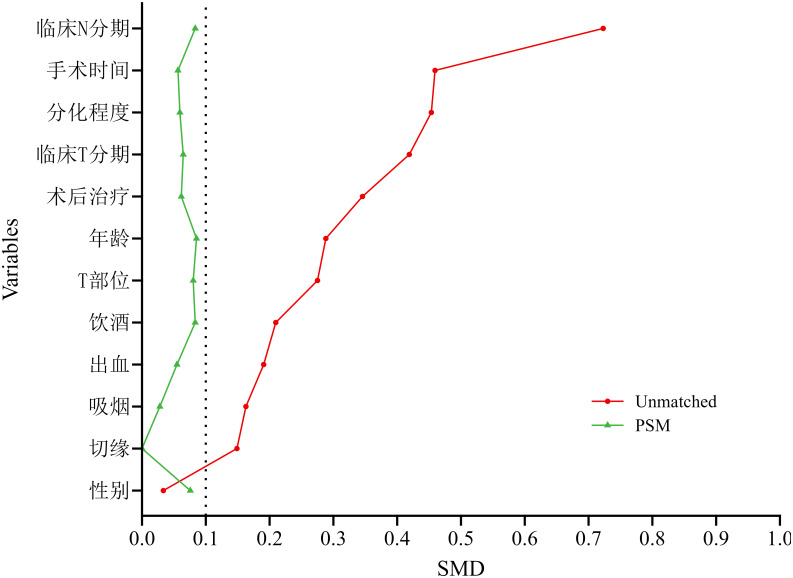
Standardized mean differences (SMDs) of propensity-score covariates. After 1:1 PSM, excellent balance was achieved between the neoadjuvant and non-neoadjuvant groups; the SMD for every covariate was < 0.1.

After matching, 144 patients were allocated to the neoadjuvant immunochemotherapy group and 144 to the upfront-surgery group. The matched sample comprised 201 men (69.8%) and 87 women (30.2%). No significant between-group differences remained for age, alcohol use, clinical T stage, clinical N stage, operative time, blood loss, differentiation, or post-operative therapy (all P > 0.05). Standardized mean differences (SMD) for all covariates were < 0.1, indicating excellent balance and effective reduction of observational bias. Detailed balance diagnostics are reported in **Table 6**, **Figure 4**.

**Table 6:** Baseline characteristics after 1:1 propensity-score matching (PSM).

Variables	Total (N = 288)	Neoadjuvant (N = 144)	Non-neoadjuvant (N = 144)	Statistic	P-value
Sex (%)				χ²=0.412	0.521
Female	87 (30.2)	46 (31.9)	41 (28.5)		
Male	201 (69.8)	98 (68.1)	103 (71.5)		
Age, Mean ± SD	65.91 ± 7.31	65.60 ± 7.52	66.22 ± 7.11	t=-0.725	0.469
Smoking (%)				χ²=0.056	0.813
No	128 (44.4)	65 (45.1)	63 (43.8)		
Yes	160 (55.6)	79 (54.9)	81 (56.2)		
Alcohol consumption (%)				χ²=0.500	0.479
No	146 (50.7)	76 (52.8)	70 (48.6)		
Yes	142 (49.3)	68 (47.2)	74 (51.4)		
Tumor location (%)				χ²=0.463	0.793
Upper	21 (7.3)	9 (6.2)	12 (8.3)		
Middle	196 (68.1)	99 (68.8)	97 (67.4)		
Lower	71 (24.7)	36 (25)	35 (24.3)		
Clinical T stage (%)				χ²=0.300	0.584
T3-T4	274 (95.1)	138 (95.8)	136 (94.4)		
T1-T2	14 (4.9)	6 (4.2)	8 (5.6)		
Clinical N stage (%)				χ²=0.500	0.479
N0	148 (51.4)	71 (49.3)	77 (53.5)		
N1	140 (48.6)	73 (50.7)	67 (46.5)		
Operation time, Median (Q1,Q3)	250.00 (220.00, 290.00)	250.00 (218.75, 300.00)	250.00 (220.00, 280.00)	z=0.441	0.659
Blood loss, Median (Q1,Q3)	100.00 (100.00, 100.00)	100.00 (100.00, 100.00)	100.00 (100.00, 100.00)	z=0.943	0.346
Differentiation (%)				χ²=0.253	0.881
Poor	63 (21.9)	33 (22.9)	30 (20.8)		
Moderate	187 (64.9)	93 (64.6)	94 (65.3)		
Well	38 (13.2)	18 (12.5)	20 (13.9)		
Margin (%)				χ²=0.000	1.000
Negative	276 (95.8)	138 (95.8)	138 (95.8)		
Positive	12 (4.2)	6 (4.2)	6 (4.2)		
Adjuvant Treatment				χ²=0.273	0.601
Observation or Chemotherapy	206 (71.5)	105 (72.9)	101 (70.1)		
Radiotherapy or Chemoradiotherapy	82 (28.5)	39 (27.1)	43 (29.9)		

After 1:1 propensity-score matching (144 pairs), all baseline characteristics were well-balanced (all P>0.05).

**Figure 5:**
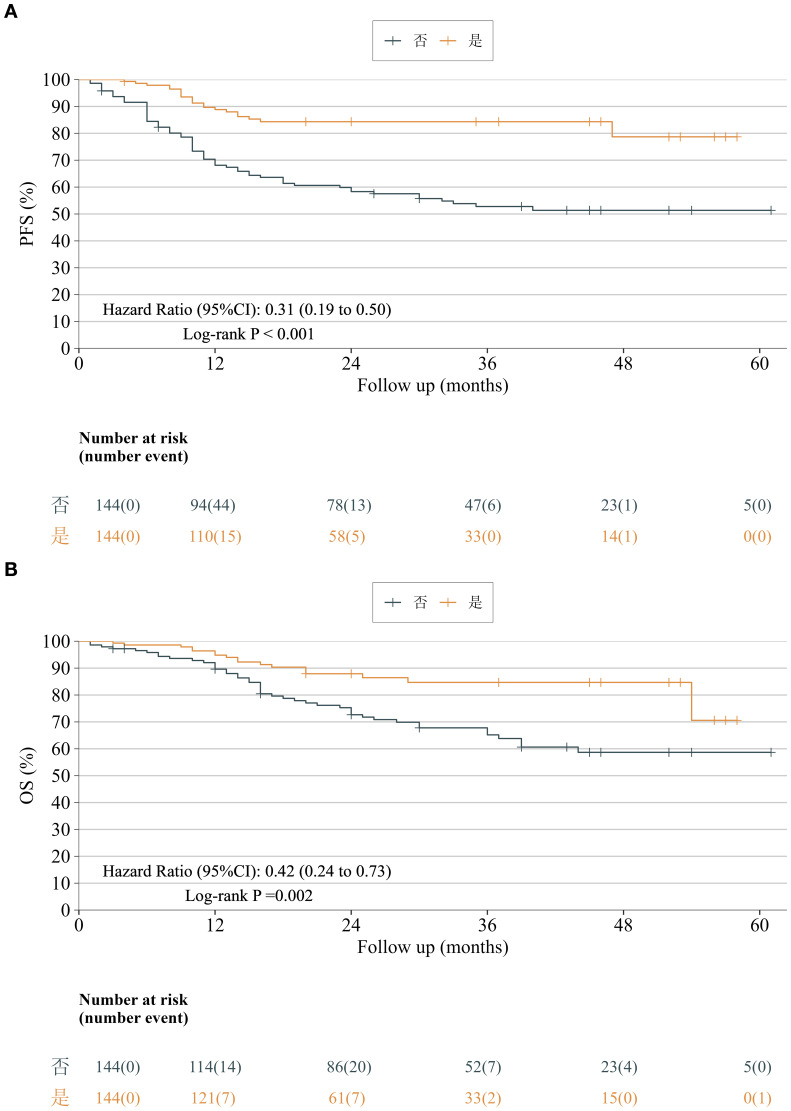
**(A)** Progression-free survival after PSM. Kaplan–Meier curves showed a significant difference in progression-free survival between the neoadjuvant-therapy and upfront-surgery groups (HR = 0.31, 95% CI 0.19–0.50, P < 0.001), corresponding to a 69% reduction in the risk of progression or death with neoadjuvant treatment. **(B)** Overall survival after PSM. Kaplan–Meier analysis revealed a significant difference in overall survival between the neoadjuvant-therapy and upfront-surgery groups (HR = 0.42, 95% CI 0.24–0.73, P = 0.002), corresponding to a 58% reduction in the risk of death with neoadjuvant treatment.

Survival analyses revealed significantly prolonged progression-free survival (PFS) in the neoadjuvant immunochemotherapy arm (HR = 0.31, 95% CI: 0.19–0.50, P < 0.001), corresponding to a 69% reduction in recurrence or progression risk. One- and three-year PFS rates were 88.8% and 84.3% versus 68.1% and 52.8% in the upfront-surgery group, respectively (**Figure 5A**).

Similarly, overall survival (OS) was markedly improved with neoadjuvant immunochemotherapy (HR = 0.42, 95% CI: 0.24–0.73, P = 0.002), translating to a 58% reduction in death risk. One- and three-year OS rates were 94.8% and 84.7% in the neoadjuvant group versus 89.6% and 65.2% in the direct surgery group (**Figure 5B**).

### Survival analysis after overlap weighting

To further mitigate baseline confounding, we applied overlap weighting (OW) based on the propensity score. The weighting model included sex, age, smoking, alcohol consumption, tumor location (T-site), clinical T stage, clinical N stage, operative time, intra-operative blood loss, differentiation, margin status, and post-operative therapy. Pathologic TNM stage was again excluded because it mediates approximately two-thirds of the neoadjuvant effect.

**Figure 6:**
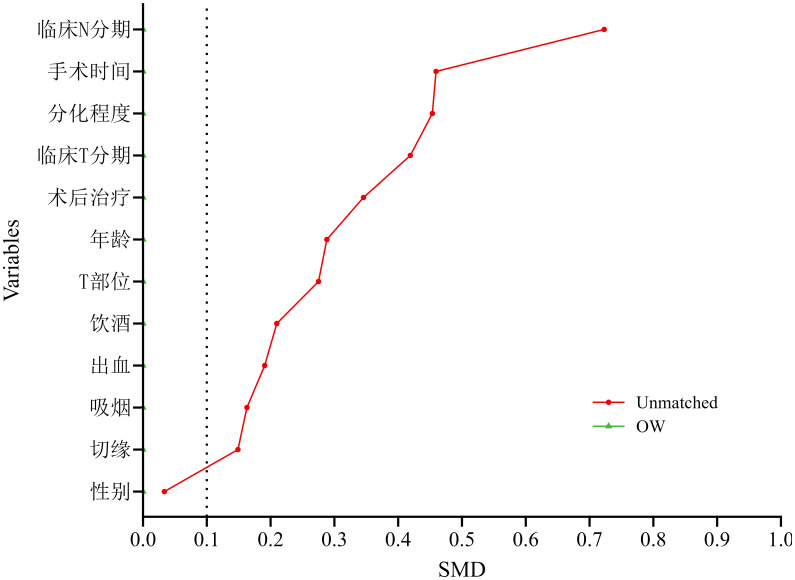
Standardized mean differences (SMDs) after overlap weighting. Following overlap weighting (OW) based on the propensity score, excellent balance was achieved between the neoadjuvant and non-neoadjuvant groups, with the SMD for every covariate approaching zero.

After OW, 97 patients remained in each arm (neoadjuvant immunochemotherapy vs. upfront surgery). The weighted cohort comprised 136 men (70.0%) and 58 women (30.0%); 103 patients (53.3%) consumed alcohol and 91 (46.7%) did not. No significant between-group differences persisted for age, alcohol use, clinical T stage, clinical N stage, operative time, blood loss, differentiation, or post-operative therapy (all P > 0.05). Standardized mean differences (SMD) for every covariate approximated zero, confirming excellent balance and minimal residual confounding (**Table 7**, **Figure 6**).

**Table 7:** Baseline characteristics after overlap weighting (OW).

Label	Stat_0	Stat_1	Stat_2	P-value
Characteristic	Overall N = 193	Neoadjuvant N = 97	Non-Neoadjuvant N = 97	P-value
Sex				>0.999
Female	57.9 (30.0)	29.0 (30.0)	29.0 (30.0)	
Male	135.2 (70.0)	67.6 (70.0)	67.6 (70.0)	
Age	65.66 ± 7.50	65.66 ± 7.52	65.66 ± 7.49	0.939
smoking				>0.999
No	90.3 (46.8)	45.1 (46.8)	45.1 (46.8)	
Yes	102.8 (53.2)	51.4 (53.2)	51.4 (53.2)	
Alcohol consumption				>0.999
No	102.9 (53.3)	51.5 (53.3)	51.5 (53.3)	
Yes	90.2 (46.7)	45.1 (46.7)	45.1 (46.7)	
Tumor location				>0.999
Upper	18.7 (9.7)	9.4 (9.7)	9.4 (9.7)	
Middle	130.4 (67.5)	65.2 (67.5)	65.2 (67.5)	
Lower	44.0 (22.8)	22.0 (22.8)	22.0 (22.8)	
Clinical T stage				>0.999
T3-T4	181.4 (93.9)	90.7 (93.9)	90.7 (93.9)	
T1-T2	11.8 (6.1)	5.9 (6.1)	5.9 (6.1)	
Clinical T stage				>0.999
N0	105.2 (54.5)	52.6 (54.5)	52.6 (54.5)	
N1	87.9 (45.5)	44.0 (45.5)	44.0 (45.5)	
Operation time	250.00 (220.00, 295.00)	250.00 (220.0,300.0)	248.00 (219.00, 280.00)	0.701
Blood loss	100.00 (100.00, 100.00)	100.00 (100.0, 100.0)	100.00 (100.00, 100.00)	0.162
Differentiation				>0.999
Poor	41.2 (21.3)	20.6 (21.3)	20.6 (21.3)	
Middle	122.5 (63.4)	61.2 (63.4)	61.2 (63.4)	
Well	29.5 (15.3)	14.7 (15.3)	14.7 (15.3)	
Margin				>0.999
Negative	185.0 (95.8)	92.5 (95.8)	92.5 (95.8)	
Positive	8.1 (4.2)	4.1 (4.2)	4.1 (4.2)	
Adjuvant Treatment				>0.999
Observation or Chemotherapy	138.5 (71.7)	69.2 (71.7)	69.2 (71.7)	
Radiotherapy or Chemoradiotherapy	54.7 (28.3)	27.3 (28.3)	27.3 (28.3)	

After OW weighting, the standardized mean differences (SMDs) for all covariates were close to zero, indicating that the overlap-weighting approach effectively achieved balance in baseline characteristics between the groups.

**Figure 7:**
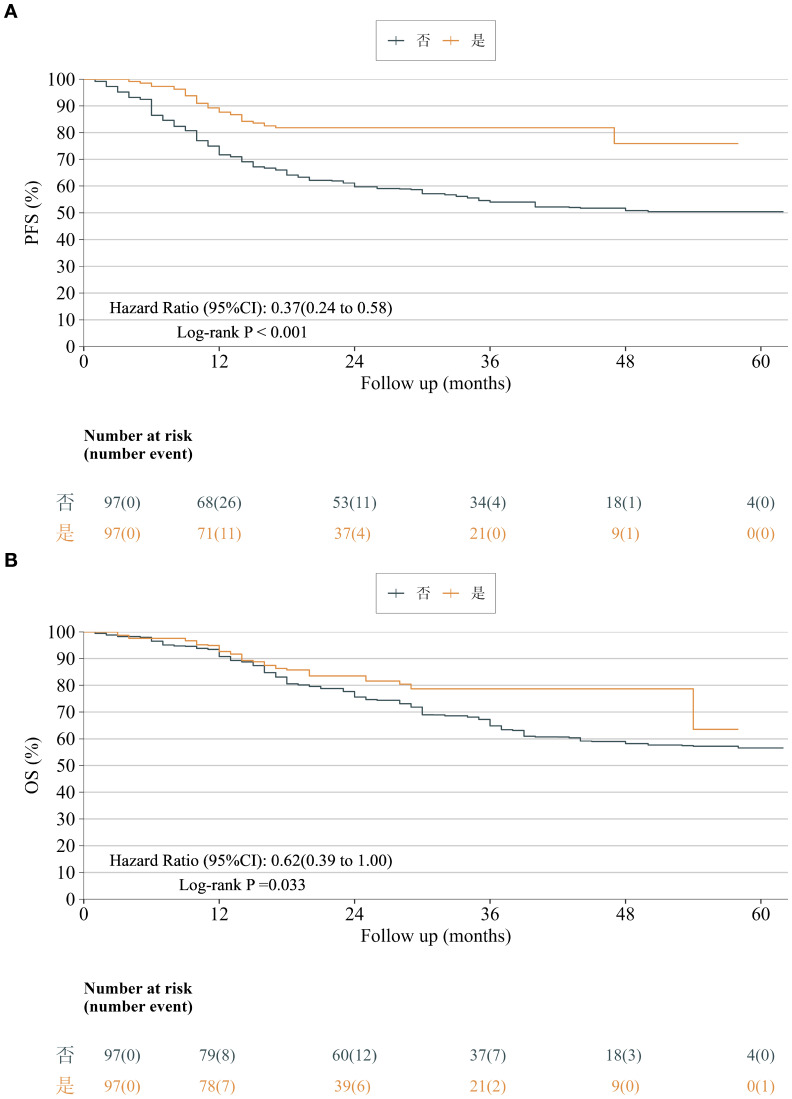
**(A)** Progression-free survival after overlap weighting. Kaplan–Meier analysis showed a significant difference in progression-free survival between the neoadjuvant-therapy and upfront-surgery groups (HR = 0.37, 95% CI: 0.24–0.58, P < 0.001), corresponding to a 63% reduction in recurrence risk with neoadjuvant treatment. **(B)** Overall survival after overlap weighting. Kaplan–Meier analysis showed a significant difference in overall survival between the neoadjuvant-therapy and upfront-surgery groups (HR = 0.62, 95% CI: 0.39–1.00, P = 0.033), corresponding to a 38% reduction in death risk with neoadjuvant treatment.

In the weighted analysis, progression-free survival (PFS) remained significantly longer with neoadjuvant immunochemotherapy (HR = 0.37, 95% CI: 0.24–0.58, P < 0.001), corresponding to a 63% reduction in recurrence or progression risk. One- and three-year PFS rates were 87.6% and 81.8% in the neoadjuvant group versus 71.7% and 54.0% in the upfront-surgery group (**Figure 7A**).

Overall survival (OS) was also improved with neoadjuvant therapy (HR = 0.62, 95% CI: 0.39–1.00, P = 0.033), translating to a 38% reduction in death risk. One- and three-year OS rates were 92.7% and 78.7% versus 90.7% and 64.8%, respectively (**Figure 7B**).

## Discussion

Esophageal cancer ranks among the most prevalent malignant neoplasms globally, with a particularly high incidence in China. The contemporary standard of care for patients with resectable esophageal cancer entails neoadjuvant radiochemotherapy (nRCT) followed by surgical resection (9, 10). However, the application of nRCT in locally advanced esophageal cancer patients remains limited due to the potential increase in surgical complexity and the risk of postoperative complications. In recent years, the burgeoning clinical evidence supporting the efficacy of immunotherapy in esophageal cancer has paved the way for the feasibility of neoadjuvant immunotherapy in resectable esophageal cancer (EC) patients.

Immune checkpoint inhibitors (ICIs) have garnered significant attention and demonstrated substantial progress in the treatment of esophageal cancer. For instance, the KEYNOTE-181 study revealed that, in the second-line treatment of advanced esophageal cancer, pembrolizumab significantly prolonged overall survival (OS) compared with chemotherapy (median OS: 6.7 months vs. 9.3 months), increased the objective response rate (ORR) (7.4% vs. 16.7%), and exhibited a lower incidence of grade 3–5 adverse events (AE) (40.9% vs. 18.2%) (3). Consistent findings have been reported in other studies, such as RATIONALE-302, ATTRACTION-3, and ESCORT, which collectively demonstrated that patients receiving immunotherapy exhibited significantly longer survival durations and higher ORRs compared with those receiving chemotherapy, along with more durable antitumor responses (11–13).

The latest results from the JUPITER-06 study indicated that toripalimab in combination with chemotherapy significantly improved progression-free survival (PFS) compared with placebo plus chemotherapy (hazard ratio, HR = 0.58; 95% confidence interval [CI], 0.46-0.74; P < 0.0001),The interim analysis of OS also showed that patients treated with toripalimab in combination with paclitaxel and cisplatin (TP) had significantly better OS compared with those treated with placebo plus TP (HR = 0.58; 95% CI, 0.43-0.78; P = 0.0004) (14). Similar findings were reported in studies such as CheckMate 648, ORIENT-15, ESCORT-1, and KEYNOTE-590, which collectively confirmed that combining programmed death protein 1 (PD-1) inhibitors with chemotherapy as a first-line treatment regimen significantly improved OS and PFS in patients with advanced EC compared with chemotherapy alone (4, 15–17). These results collectively suggest that ICIs hold broad application prospects in the first-line treatment of advanced EC.

When compared with adjuvant immunotherapy, neoadjuvant immunotherapy has demonstrated potential advantages in both theoretical and practical aspects (18–20). A study conducted in 2016 using a spontaneous metastatic breast cancer mouse model revealed that neoadjuvant immunotherapy significantly enhanced the treatment outcomes for distant metastases after primary tumor resection compared with adjuvant immunotherapy, particularly in eradicating distant microscopic metastases (0/5 vs. 3/5) (21).

In the multicenter, open-label phase III NEOCRTEC5010 trial (n = 451), Yang and colleagues demonstrated that CRT-S significantly prolonged survival over surgery alone (S) in patients with locally advanced esophageal squamous cell carcinoma (ESCC). The 3- and 5-year progression-free survival (PFS) rates were 69% and 64% in the CRT-S group versus 50% and 43% in the S group (HR 0.58; 95% CI 0.43–0.79; P < 0.001). Similarly, 3- and 5-year overall survival (OS) were 66% and 59% with CRT-S compared with 60% and 49% with S alone (HR 0.74; 95% CI 0.56–0.97; P = 0.03), establishing CRT-S as a standard-of-care in this population (22). The Japanese JCOG1109 NExT phase III trial (n = 501) compared three neoadjuvant strategies: DCF(Docetaxel, Cisplatin, and 5-Fluorouracil) triplet chemotherapy, CF(Cisplatin and 5-Fluorouracil) doublet chemotherapy, and CF doublet plus radiotherapy (CRT-S). DCF achieved superior 3-year PFS (61.8%) and OS (72.1%) versus both CF (PFS 47.7%, OS 62.6%) and CF+RT (PFS 57.3%, OS 68.1%); no significant differences were observed between CF and CF+RT. These data indicate that DCF represents an effective alternative to CRT-S, although a direct head-to-head comparison is lacking (23). In our single-institution retrospective cohort (n = 623), Neoadjuvant immunochemotherapy followed by surgery(ICT-S) yielded 1- and 3-year PFS rates of 88% and 82%, respectively, compared with 68% and 51% for upfront surgery (HR 0.37; 95% CI 0.22–0.61; P < 0.0001). Corresponding OS rates were 95% and 84% versus 90% and 61% (HR 0.46; 95% CI 0.29–0.73; P = 0.0005); 5-year OS remains immature. Although these early survival outcomes appear non-inferior to CRT-S and DCF, the non-randomized design mandates cautious interpretation and underscores the need for prospective phase III validation.

However, the present study has several limitations. First, as a non-randomized clinical trial, it is inherently susceptible to selection bias. To mitigate this as much as possible, propensity score matching (PSM) and overlap weighting (OW) were used to balance patient baseline clinical characteristics, and a unified surgical team was implemented to enhance the reliability of the results. Additionally, although the number of dropouts was relatively small (12/635, 1.9%) and primarily due to loss to follow-up or withdrawal of consent, this attrition may still have introduced non-negligible bias into the final efficacy evaluation.

## Conclusions

For patients with locally advanced esophageal cancer, the combination of immunotherapy and chemotherapy as a neoadjuvant treatment regimen is effective. The results of this study confirmed that surgical resection following preoperative neoadjuvant immunotherapy combined with chemotherapy is safe and does not increase the risk of postoperative complications. Compared with the direct surgery group, this regimen significantly improves PFS and OS after surgery, thereby providing novel insights and evidence for the treatment of locally advanced esophageal cancer.

## Data Availability

The raw data supporting the conclusions of this article will be made available by the authors, without undue reservation.
