# Supplementary material for: Efficacy of neoadjuvant immunochemotherapy in locally advanced esophageal squamous cell carcinoma: a prospective cohort study with propensity-score matching
Source: Front Immunol. 2026 Jan 6;16:1711095. doi: 10.3389/fimmu.2025.1711095 (PMC12816230; doi:10.3389/fimmu.2025.1711095)
Supplement: Supplementary file 1 [file Table1.docx]

Adverse Event (Patients=192) Any Grade, n (%) Grade 1-2 Grade 3-4

Leukopenia 76 (39.6) 72 4

Thrombocytopenia 48 (25.0) 46 2

Abnormal liver function 37 (19.3) 37 0

Abnormal kidney function 41 (21.3) 38 3

Hypothyroidism 27 (14.1) 27 0

Diabetes mellitus 2 (1.0) 1 1

Hypophysitis 3 (1.6) 1 2

Immune-mediated pneumonia 1 (0.5) 0 1

Neutropenia 39 (20.3) 38 1

Rash 33 (17.2) 30 3

Alopecia 133 (69.3) 133 0

Nausea 111 (57.8) 111 0

Vomiting 33 (17.2) 31 2

Fatigue 27 (14.1) 27 0

Peripheral neuritis 11 (5.7) 11 0

Diarrhea and colitis 6 (3.1) 5 1

Hyperthyroidism 6 (3.1) 6 0
